# A cross-lagged prospective network analysis of depression and anxiety and cognitive functioning components in midlife community adult women

**DOI:** 10.1017/S0033291722000848

**Published:** 2023-07

**Authors:** Nur Hani Zainal, Michelle G. Newman

**Affiliations:** Department of Psychology, The Pennsylvania State University, University Park, Pennsylvania, United States

**Keywords:** Anxiety, cross-lagged network analysis, depression, interpersonal, scar theory

## Abstract

**Background:**

Scar theory proposes that heightened depression and anxiety precede and predict worse cognitive functioning outcomes, whereas the vulnerability theory posits the opposite pathway. However, most investigations on this topic have been cross-sectional, precluding causal inferences. Thus, we used cross-lagged prospective network analyses to facilitate causal inferences in understanding the relations between psychopathology and cognitive functioning components.

**Methods:**

Racially-diverse midlife women (*n* = 1816) participated in the Study of Women's Health Across the Nation at two time-points, spanning one year apart. Five psychopathology (anxiety severity, depressed mood, somatic symptoms, positive affect, interpersonal problems) and four cognitive functioning nodes (working memory (WM), processing speed (PS), facial recognition (FCR), and verbal memory (VRM)) were assessed. All analyses adjusted for age, menopausal status, estradiol, and follicle-stimulating hormones.

**Results:**

Contemporaneous networks yielded notable inverse between-node relations (*edges*) between interpersonal problems and reduced FCR and PS, and between depressed mood and lower FCR, VRM, or PS. Nodes that had the highest likelihood to bridge other constructs were positive affect, anxiety severity, WM, and VRM. Temporal networks produced edges consistent with the scar (*v*. vulnerability) hypotheses. Higher somatic symptoms were related to reduced PS and WM, and greater depressed mood was correlated with lower future PS and WM. Also, higher anxiety severity coincided with decreased future PS and WM. Greater positive affect was associated with stronger future PS, FCR, and WM. Also, positive affect had the strongest relations with other nodes.

**Conclusions:**

Findings suggest the importance of targeting symptoms and cognitive functioning simultaneously.

Everyday life activities, such as reading, exercising, and planning, require crucial aspects of executive functioning, such as working memory (WM). WM is defined as the ability to register, preserve, and alter cognitive representations of incoming data online (Schmank, Goring, Kovacs, & Conway, 2019). Sustaining intact WM is essential as it is intrinsically tied to other vital cognitive abilities. These include attention, verbal memory (capacity to retain and retrieve verbal material, events, or facts stored in long-term memory), processing speed (degree of efficiency toward completion of a task-at-hand), and social cognition (e.g. facial recognition) (Chen, Norton, McBain, Ongur, & Heckers, 2009). Notably, these cognitive functioning domains cohere together, mutually reinforce one another, and relate to optimal engagement in myriad cognitive and behavioral processes (Schmank et al., 2019). For example, these cognitive functioning components facilitate effective strategizing, problem-solving, managing feelings, taking reasonable risks, resolving disputes, and implementing other goal-directed activities in career and relationship contexts (Abramovitch, Short, & Schweiger, 2021). Thus, understanding the predictors, correlates, and outcomes of reduced WM, verbal memory, facial recognition, and processing speed is essential.

Scar theories postulate that heightened depression, anxiety, and their accompanying interpersonal deficits may be precursors and correlates of weakened cognitive functioning capacities (Clayton, Giletta, Boettiger, & Prinstein, 2021; Zainal & Newman, 2022). This ‘scarring’ relation may occur via the buildup of stress-linked biomarkers (e.g. inflammation) and increased long-term wear-and-tear of the hypothalamic-pituitary-adrenal axis and related neurophysiological systems implicated for WM, processing speed, and verbal memory abilities. Other possible factors that mediate psychopathology–future cognitive dysfunction relations include non-constructive thinking patterns, reduced stress endurance, and increased negative affect (Burcusa & Iacono, 2007). Conversely, vulnerability theories posit that reduced cognitive functioning domains precede and relate to future elevated anxiety, depressive somatic/vegetative symptoms, diminished positive affect, and more relationship issues (Romer & Pizzagalli, 2021; Zainal & Newman, 2021a). Lower cognitive functioning capacities could correlate with worse future levels of anxiety and depression through low stress or uncertainty tolerance, avoidance of negative emotional shifts, decreased goal-directed actions, and biases toward and difficulties detaching from negativity (Bernstein, Heeren, & McNally, 2017; Newman et al., 2019).

Substantiating scar and vulnerability theories, a recent systematic quantitative synthesis that pooled data across 82 meta-analyses (Abramovitch et al., 2021) showed that heightened depression, anxiety, and related disorders were associated with cognitive dysfunction. Evidence also exists that associations between higher depression components (e.g. symptom severity, interpersonal issues) and greater cognitive dysfunction occurred 2–9 years later in large community samples (e.g. Clayton et al., 2021; Zainal & Newman, 2021a). Moreover, such links between increased anxiety and depression indicators and higher cognitive dysfunction were replicated across various cultures, with mounting evidence that the scarring relation was pronounced in women (e.g. Barak, Barson, Davie, Glue, & Paleacu, 2021).

Nonetheless, most prospective investigations on cognitive dysfunction–depression and anxiety relations thus far have used ordinary least squares regression or structural equation modeling (SEM). These approaches tend to provide only one global (*v*. specific) cognitive dysfunction–depression and anxiety association. In addition, SEM presumes that components (or indicators) of depression and anxiety passively reflect a latent global construct (cf. local independence assumption), instead of allowing for these components to relate to one another in a mutually reinforcing way (Schmank et al., 2019). Cross-lagged panel network analysis (CLPN) (Epskamp, 2020) was thus developed to permit the distinguishment of components (known as *nodes*) of common mental health problems and their scar/vulnerability factor components when examining their relations. Also, CLPN and SEM produce dissimilar models that lead to different theoretical and applied inferences (van Bork et al., 2021). Similar to performing several multivariate linear regressions simultaneously, CLPN yields regularized partial correlations between nodes (also called *edges*) that adjust for other edges in the network. In the process, it refines our understanding of cognitive dysfunction–psychopathology relations. This is important for clinical science as knowledge of the unique node in a cognitive functioning cluster that has the strongest relations/edges with nodes in a psychopathology cluster (and *vice versa*) informs the value of augmenting evidence-based treatments (e.g. cognitive–behavioral therapies). Thus, it may guide the development of novel cognitive functioning interventions for depression and anxiety disorders (Therond et al., 2021). Further, the translational implications of the current CLPN study can be applied at both preventative and treatment stages. Notably, using CLPN aligns with the mission of precision psychiatry by determining if and how unique cognitive functioning nodes *bridge* between depression and anxiety constructs during one time-point and across multiple time-points (Epskamp, Borsboom, & Fried, 2018). Moreover, applying this network perspective is essential given the increasing prevalence and burden of neuropsychiatric diseases worldwide, such as major depression and various dementia syndromes (Ettman et al., 2020; Wolters et al., 2020).

As yet, nine cross-sectional studies across diverse cultures have examined the contemporaneous network relations between depression and performance-based cognitive functioning nodes. Patients with unipolar depression (*v*. bipolar disorder; BD) showed a denser cognitive functioning network, indicating more significant pathology (Galimberti et al., 2020). Also, verbal memory was the most influential node in unipolar depression, but not BD (Galimberti et al., 2020). Similarly, higher depression and more frequent repetitive negative thinking correlated with reduced WM, verbal memory, global cognition, and language among young and midlife community adults (Bernstein et al., 2019; Jia et al., 2020; Jiang et al., 2022), but not remitted depressed patients (Hoorelbeke, Marchetti, De Schryver, & Koster, 2016). Also, WM and inhibition were key EF components that bridged across internalizing symptoms (e.g. anxiety, depression) and externalizing symptoms (e.g. irritability) in youth with ADHD (Eadeh, Markon, Nigg, & Nikolas, 2021) and eating disorders (Byrne et al., 2021). Further, deficits in expressivity (e.g. blunted affect) co-occurred with worse global cognition and processing speed among community adults with schizophrenia (Galderisi et al., 2018) and patients with first-episode psychosis (Chang et al., 2020).

Despite their informative value to comprehend psychopathology–cognitive functioning relations at one time-point, these cross-sectional network analysis studies preclude causal inferences. To the best of our awareness, there have only been two prospective network analyses on this topic thus far. One study (van Wanrooij, Borsboom, Moll van Charante, Richard, & van Gool, 2019) found that self-reported memory issues on a depression scale correlated with subsequent dementia; however, the reverse relation was not tested, and no behavioral cognitive functioning tests were administered. Another study (Zainal & Newman, 2021b) observed that anxiety and depression (*v*. seven other psychopathology components such as aberrant motor behaviors, hallucinations, delusions, etc.) had the largest relation to executive dysfunction measured about 2 years later.

Hence, the current study used CLPN to elucidate the relations between five nodes of depression and anxiety (depressed mood, interpersonal problems, low positive affect, somatic symptoms, anxiety severity) and four cognitive functioning nodes (WM, verbal memory, facial recognition, processing speed). We hypothesized that contemporaneous networks would show non-zero estimated negative edges between depression and anxiety and cognitive functioning nodes. Based on the most up-to-date meta-analysis (Abramovitch et al., 2021) and a recent CLPN study on this topic (Zainal & Newman, 2021b), we also predicted non-zero estimated edges indicating that higher depression and anxiety nodes would relate to reduced cognitive functioning (scar theory), as opposed to the reverse direction (vulnerability hypothesis).

## Method

### Participants

The current study was a secondary analysis of the Study of Women's Health Across the Nation (SWAN) dataset (Greendale et al., 2010). Participants (*n* = 1816) were middle-to-older aged adults at Wave 1 (W1; *M* age = 53.28 years, s.d. = 2.62, range = 49–63) and Wave 2 (W2; *M* age = 54.81 years, s.d. = 2.87, range = 50–64), and 100% were female. Individuals racially self-identified as Black (26.5%), Asian (13.3%), White (44.4%), or another race (15.7%). Ethnically, whereas 15.7% identified as Hispanic, the remaining 84.3% identified as not Hispanic. Also, 40.8% attained college or post-graduate education. Online Supplementary Table S1 in the online Supplemental material details socio-demographic and related variables.

### Procedures

Participants completed a self-report measure of depression and anxiety symptom severity and face-to-face neuropsychological testing at W1 (2004–2006) and W2 (2005–2007). The two assessment waves were chosen for this secondary analysis because they contained data that addressed our research question. Before administration, cognitive functioning tests were forward and backward translated in Spanish, Cantonese, and Japanese in a valid manner (Greendale et al., 2010). Further, bilingual participants chose whether their face-to-face neuropsychological testing was in their native language or English.

### Measures

#### Depression components

The 20-item Center for Epidemiologic Studies Depression (CES-D) Scale (Cosco, Prina, Stubbs, & Wu, 2017) measured past-week depression. Respondents rated items on a five-point Likert scale from 0 = *rarely* to 4 = *most or all of the time*. It had good discriminant and convergent validity and high retest reliability (Cosco et al., 2017). In a general population of midlife-to-older adults, the CES-D was comprised of four components: depressed mood (e.g. ‘I felt depressed’); positive affect (e.g. ‘I was happy’); somatic symptoms (e.g. ‘I did not feel like eating; my appetite was poor’); and interpersonal problems (e.g. ‘I felt lonely’) (Cosco et al., 2017). An averaged score for each subscale represented a depression component node. Scores ranged from 0 to 4 with higher scores indicating more depression. Also, internal consistency scores herein were good across all time-points for all subscales (depressed mood: Cronbach's *α* = 0.937–0.943; positive affect: *α* = 0.946–0.947; somatic symptoms: *α* = 0.849–0.860; interpersonal problems: *α* = 0.711–0.739).

#### Anxiety severity

Anxiety severity was measured referencing the past 2 weeks with four items rated on a five-point Likert scale ranging from 1 = *not at all* to 5 = *daily*. Items included fearfulness without areason, accelerated heart rate/pounding heart, irritability/grouchiness, and feeling nervous/tense (Bromberger et al., 2013). A mean score representing an anxiety node was calculated across both time-points. Scores ranged from 1 to 5 with higher scores indicating greater anxiety. Scores had good internal consistency (*α* = 0.868–0.886), discriminant validity with the CESD (e.g. *r* = 0.57) (Kravitz, Schott, Joffe, Cyranowski, & Bromberger, 2014), and convergent validity with the GAD-7 (*r* = 0.71) (Bromberger et al., 2013), a measure of general anxiety (Spitzer, Kroenke, Williams, & Löwe, 2006).^†^^1^

#### Processing speed

The Symbol Digit Modalities Test (SDMT) (Pereira, Costa, & Cerqueira, 2015) measured processing speed. Respondents had to pair unique symbols with specific numbers within 1.5 minutes. Possible SDMT scores ranged from 0 to 110, with higher scores indicating faster processing speed. The SDMT has shown good internal consistency, high retest reliability, strong convergent validity, and good discriminant validity (e.g. low correlations with scores on basic and higher-order attention tests; Bates & Lemay, 2004; Pereira et al., 2015).

#### Verbal memory

The East Boston Memory Test (EBMT; Albert et al., 1991) assessed verbal memory by asking participants to recall 12 details of a 36-word paragraph story following a 10 min delay period. Possible EBMT scores ranged from 0 to 12, with higher scores representing better verbal memory. It has shown excellent retest reliability, good construct validity, and strong discriminant validity (Albert et al., 1991). Internal consistency was good in the present study (*α* = 0.981–0.982 in the current study).

#### Face recognition

Facial recognition was evaluated using the 48-item Wechsler Memory Scale-III Faces – delayed recall scale (Wechsler, 1997). Participants were shown 48 faces (24 targets and 24 distractors, each displayed for 1 s) and were tested on their ability to recall them after a 30 min delay. Possible scores ranged from 0 to 48, with higher scores suggesting stronger facial recognition. It has demonstrated strong retest reliability, high convergent validity (Wechsler, 1997), and strong discriminant validity among patients with and without Alzheimer's disease (Seelye, Howieson, Wild, Moore, & Kaye, 2009). Also, it has shown strong internal consistency across time (*α* = 0.995–0.996 herein).

#### Working memory

WM was measured with the backward digit span, in which participants repeated increasingly longer number strings ranging from 2 to 7 in reverse order, with each string length comprising two trials (Psychological Corporation, 1997). Possible scores ranged from 0 to 12, with higher scores indicating stronger WM. This test has shown excellent internal consistency, good retest reliability, and strong convergent and discriminant validity (Psychological Corporation, 1997). Its scores also have good internal consistency at both time-points (*α* = 0.953–0.968 herein).^2^

### Statistical analysis

All analyses were conducted in *R* Version 4.1.0 and *RStudio* Version 1.4.1717 (R Core Team, 2021). Missing data (comprising 29.2% of total observations) were managed using multiple imputation with the *mice R* package (van Buuren & Groothuis-Oudshoorn, 2011), a gold standard method. Data were aggregated across 10 multiply imputed datasets with iterations. Moreover, we included auxiliary variables (age, baseline menopausal status, follicle-stimulating hormone, estradiol, depression and anxiety severity, cognitive functioning) in the multiple imputation models. Compared to complete case analysis, multiple imputation produces more accurate, unbiased, and efficient parameter and standard error estimates, even with high missingness, and was appropriate based on the missing at random assumption (Lee & Shi, 2021; Madley-Dowd, Hughes, Tilling, & Heron, 2019). Also, no outliers were identified in the imputed dataset. Table 1 presents the descriptive statistics of depression, anxiety, and cognitive functioning components (raw scores) at W1 and W2.

**Table 1.** Descriptive statistics of network components

	Depressed mood	Positive affect	Somatic symptoms	Interpersonal problems	Anxiety symptoms	Verbal memory	Face recognition	Processing speed	Working memory
Wave 1									
*M*	1.459	2.412	1.612	1.229	2.146	9.647	40.241	56.680	7.288
(s.d.)	(1.396)	(1.611)	(1.122)	(1.34)	(1.237)	(2.123)	(4.761)	(13.126)	(2.487)
Min	0	0	0	0	1	4	24	1	1
Max	4	4	4	4	5	12	48	94	12
Skewness	0.424	−0.520	0.236	0.632	1.202	−0.736	−0.636	−0.892	0.124
Kurtosis	−1.393	−1.439	−1.240	−1.030	0.123	−0.252	−0.082	2.247	−0.762
Wave 2									
*M*	1.611	2.344	1.643	1.387	2.048	9.172	38.524	52.004	6.773
(s.d.)	(1.353)	(1.597)	(1.047)	(1.339)	(1.141)	(2.171)	(5.755)	(12.831)	(2.898)
Min	0	0	0	0	1	0	24	4	1
Max	4	4	4	4	5	12	48	88	12
Skewness	0.154	−0.445	0.057	0.459	1.305	−0.564	−0.419	−0.168	0.079
Kurtosis	−1.544	−1.486	−1.211	−1.170	0.484	−0.328	−0.628	−0.253	−0.879

*M*, mean; s.d., standard deviation; Min, minimum; Max, maximum.

Depressed mood, positive affect, somatic symptoms, and interpersonal problems were derived from the Center for Epidemiologic Scale for Depression and could range from 0 (*rarely*) to 4 (*most or all of the time*). All values represent raw scores.

All network analyses were performed with the *bootnet* (Epskamp et al., 2018), *glmnet* (Friedman, Hastie, & Tibshirani, 2010), *networktools* (Haslbeck & Waldorp, 2018; Jones, 2020), *psychonetrics* (Epskamp, 2020), and *qgraph* (Epskamp, Cramer, Waldorp, Schmittmann, & Borsboom, 2012) *R* packages. First, network graphs were constructed in which nodes closer to one another had higher associations with each other, and nodes positioned nearer to the center evidenced stronger relations with other nodes. Next, a graphical Gaussian model (GGM) (Epskamp et al., 2012) was used wherein edges signified relations between nodes after adjusting for the influence of all other nodes. In the process, GGMs were regularized using the least absolute shrinkage and selection operator (LASSO), which computed partial associations and removed false-positive (i.e. weak or spurious) edges by reducing them to zero. Further, the graphical LASSO was utilized with the extended Bayesian information criterion (EBIC) model selection, in which the model with the lowest EBIC value out of 100 was chosen. With this approach, the hyperparameter *γ* = 0.5 value was selected because it balanced sensitivity (i.e. eliminating true edges) and specificity (i.e. including false-positive edges), and maximized the chances that genuine edges were chosen. Also, CLPN controlled for baseline scores of all concurrently measured nodes (i.e. each unique edge accounted for W1 scores of the W2 node and all other nodes). In addition, based on literature (Berent-Spillson et al., 2012), the following variables were added to the models as covariates: age (years), follicle-stimulating hormone (mIU/mL), estradiol (pg/mL), and menopausal status (premenopausal, early perimenopausal, late perimenopausal, post-menopausal).

Next, centrality indices were calculated to determine the importance of each node (i.e. the extent to which it related to all nodes of the other cluster or construct). For contemporaneous networks, the *two-step bridge EI* was computed to elucidate the relations among depression, anxiety, and cognitive functioning components (Jones, 2020). The two-step bridge *EI* comprised bridge *EI1* (total sum of edge weights from a unique node to all nodes of the other cluster) and bridge *EI2* (bridge *EI1* factoring in the ancillary effect of a unique node through the effects of closest nodes in its vicinity). Higher bridge *EI1* and *EI2* values indicated stronger effect of nodes on the other cluster. For temporal networks, we computed the cross-construct *in-prediction* (or *predictability*; i.e. the degree to which proportion of variance of a unique node at W2 was explained by W1 nodes of the other cluster) (Haslbeck & Waldorp, 2018). In addition, we calculated the cross-construct *out-prediction* (or *influence*; i.e. the extent to which a unique W1 node accounted for the variance of all W2 nodes of the other cluster) (Haslbeck & Waldorp, 2018). Further, to determine stability of network metrics (i.e. edge strength and centrality indices – two-step bridge EI, in-prediction, out-prediction), we computed edge weights 95% confidence interval (CI) and correlation stability (CS) coefficients (Epskamp et al., 2018). CS coefficient values ≥0.25 were considered acceptable, whereas CS coefficient values ≥0.50 were regarded as good. Additionally, the data analytic scripts of the present study have been uploaded to OSF (https://osf.io/dh7nb/).

## Results

### Contemporaneous networks

**Fig. 1.**
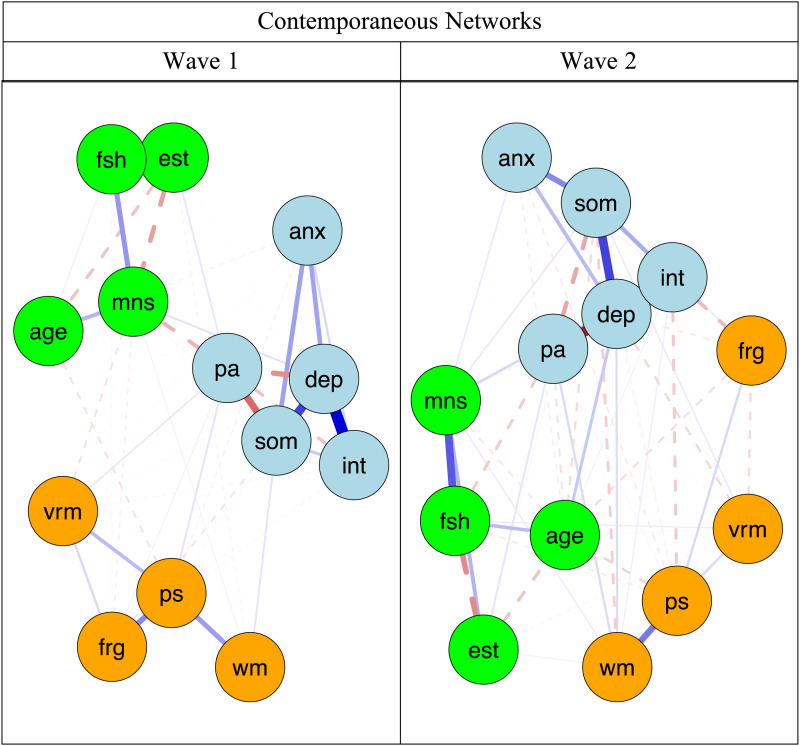
Contemporaneous networks of cognitive functioning and depression components. anx, anxiety severity; dep, depressed mood; frg, face recognition; vrm, verbal memory; int, interpersonal problems; pa, positive affect; ps, processing speed; som, somatic symptoms; fsh, follicle-stimulating hormone (mIU/mL); est, estradiol (pg/mL); age, age of participants at respective wave; mns, menopausal status (pre-menopausal, early perimenopausal, late perimenopausal, and post-menopausal). Light grey nodes indicate mental health symptoms, white nodes reflect cognitive functioning domains, and black nodes denote covariates. Black/grey lines indicate positive relations, whereas grey dotted lines signal negative relations, and line thickness and boldness reflect strength of associations.

Figure 1 displays the contemporaneous networks during W1 and W2. Blue lines indicate positive relations, whereas red dotted lines signal negative relations. Line thickness reflects strength of associations. Table 2 presents the strongest undirected edges within and across constructs. Across network clusters, the strongest non-zero estimated edges were negatively-signed interpersonal problems–face recognition (*r* = −0.074), interpersonal problems–processing speed (*r* = −0.056), depressed mood–face recognition (*r* = −0.049), depressed mood–verbal memory (*r* = −0.045), anxiety–processing speed (*r* = −0.034), and somatic symptoms–WM (*r* = −0.018). Moreover, higher positive affect was associated with stronger WM (*r* = 0.033) and processing speed (*r* = 0.033). The depression and anxiety components with the highest bridge EIs were positive affect (bridge *EI1* = 0.144, bridge *EI2* = 0.859) and anxiety severity (bridge *EI1* = −0.217, bridge *EI2* = −0.765). Within the cognitive functioning cluster, WM (bridge *EI1* = −0.032, bridge *EI2* = −0.575) and verbal memory (bridge *EI1* = −0.596, bridge *EI2* = −3.107) had the largest bridge EIs (see online Supplementary Fig. S1 for more details). Contemporaneous network metrics showed high stability for edge strength (CS = 0.750, 95% CI 0.672–1.000). In addition, bridge EI showed a strong degree of stability (0.517, 95% CI 0.439–0.595).

**Table 2.** Strongest undirected edges of contemporaneous networks

Measure	Undirected edge weight		
	W1	W2	Average
Depressed mood–positive affect	−0.220	−0.430	−0.325
Estradiol–FSH	−0.379	−0.215	−0.297
Positive affect–somatic symptoms	−0.297	−0.160	−0.228
Estradiol–age	−0.116	−0.108	−0.112
Positive affect–interpersonal problems	−0.122	−0.070	−0.096
Positive affect–menopausal status	−0.146	−0.026	−0.086
Interpersonal problems–face recognition	−0.019	−0.129	−0.074
Processing speed–age	−0.059	−0.078	−0.069
Interpersonal problems–processing speed	−0.013	−0.099	−0.056
Depressed mood–face recognition	−0.055	−0.042	−0.049
Positive affect–FSH	0.000	−0.097	−0.049
Depressed mood–verbal memory	−0.015	−0.074	−0.045
Positive affect–age	−0.015	−0.056	−0.035
Anxiety symptoms–processing speed	−0.028	−0.040	−0.034
Somatic symptoms–age	0.000	−0.067	−0.034
Face recognition–age	0.000	−0.063	−0.032
Anxiety symptoms–age	−0.027	−0.034	−0.031
Verbal memory–menopausal status	−0.059	0.000	−0.030
Estradiol–menopausal status	−0.186	0.141	−0.023
Processing speed–FSH	0.000	−0.039	−0.020
Somatic symptoms–working memory	0.054	−0.090	−0.018
Depressed mood–working memory	0.000	0.065	0.033
Positive affect–processing speed	0.055	0.011	0.033
Positive affect–working memory	0.000	0.066	0.033
Depressed mood–menopausal status	0.060	0.017	0.038
Interpersonal problems–menopausal status	0.000	0.077	0.039
Interpersonal problems–anxiety symptoms	0.074	0.019	0.047
Positive affect–estradiol	0.047	0.049	0.048
Age–menopausal status	0.149	−0.046	0.051
Depressed mood–age	0.000	0.103	0.052
FSH–age	0.026	0.124	0.075
Verbal memory–processing speed	0.135	0.067	0.101
Somatic symptoms–interpersonal problems	0.109	0.155	0.132
Face recognition–processing speed	0.234	0.072	0.153
Depressed mood–anxiety symptoms	0.184	0.126	0.155
Somatic symptoms–anxiety symptoms	0.188	0.219	0.204
Processing speed–working memory	0.198	0.251	0.224
FSH–menopausal status	0.209	0.310	0.259
Depressed mood–somatic symptoms	0.377	0.353	0.365
Depressed mood–interpersonal problems	0.506	0.473	0.490

FSH, follicle-stimulating hormone (mIU/mL); W1, wave 1; W2, wave 2.

### Temporal networks

**Fig. 2.**
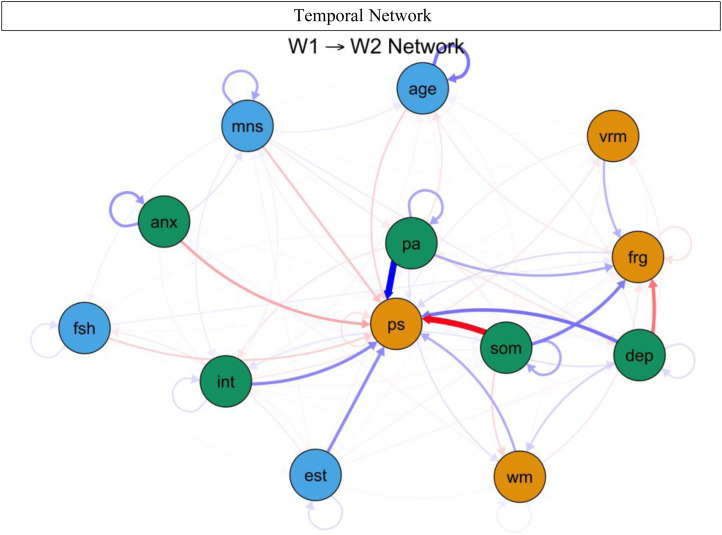
Temporal network of cognitive functioning and depression components. anx, anxiety severity; dep, depressed mood; frg, face recognition; vrm, verbal memory; int, interpersonal problems; pa, positive affect; ps, processing speed; som, somatic symptoms; fsh, follicle-stimulating hormone (mIU/mL); est, estradiol (pg/mL); age, age of participants at respective wave; mns, menopausal status (pre-menopausal, early perimenopausal, late perimenopausal, and post-menopausal). White nodes indicate mental health symptoms, black nodes reflect cognitive functioning domains, and dark grey nodes denote covariates. Black/grey lines indicate positive relations, whereas grey dotted lines signal negative relations, and line thickness and boldness reflect strength of associations; W1, wave 1; W2, wave 2.

**Fig. 3.**
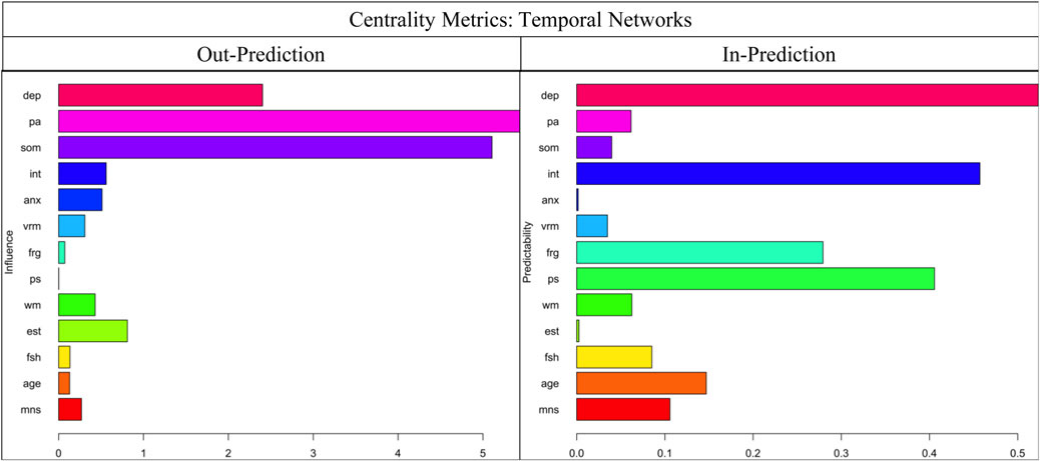
In-prediction and out-prediction of temporal network. anx, anxiety severity; dep, depressed mood; frg, face recognition; vrm, verbal memory; int, interpersonal problems; pa, positive affect; ps, processing speed; som, somatic symptoms; fsh, follicle-stimulating hormone (mIU/mL); est, estradiol (pg/mL); age, age of participants at respective wave; mns, menopausal status (pre-menopausal, early perimenopausal, late perimenopausal, and post-menopausal). White bars indicate mental health symptoms, black bars reflect cognitive functioning domains, and grey bars denote covariates.

Figure 2 shows the CLPN, with arrows relaying temporal associations of the edges within and across constructs. Nodes with the greatest auto-regression coefficients were age (*r* = 0.996), anxiety severity (*r* = 0.770), and positive affect (*r* = 0.647) (refer to online Supplementary Fig. S2). Table 3 displays the strongest directed edges within and across constructs or clusters. Across clusters, higher W1 somatic symptoms were related to lower W2 processing speed (*d* = −3.598) and WM (*d* = −0.253). Also, greater W1 depressed mood was associated with decreased W2 face recognition (*d* = −0.997) and verbal memory (*d* = −0.117). In addition, higher anxiety severity correlated with reduced W2 processing speed (*d* = −0.596) and WM (*d* = −0.089). Other cross-construct edges that emerged included W1 interpersonal problems–W2 verbal memory (*d* = −0.153) and W1 interpersonal problems–W2 face recognition (*d* = −0.058). Moreover, higher W1 positive affect was related to stronger W2 processing speed (*d* = 9.583), face recognition (*d* = 0.558), and WM (*d* = 0.173). Additionally, no true edges displaying negative relations between W1 cognitive functioning and W2 depression components emerged. As shown in Fig. 3, across clusters, the most impactful nodes with high out-prediction and low in-prediction values were positive affect (*β* = 5.440) and somatic symptoms (*β* = 5.106), and the least influential nodes with low out-prediction and high in-prediction values were depressed mood (*β* = 0.523) and processing speed (*β* = 0.457). Moreover, temporal network metric coefficients showed strong stability for edge strength (CS = 0.672, 95% CI 0.595–1.000), in-prediction (CS = 0.672, 95% CI 0.595–1.000), and out-prediction (CS = 0.672, 95% CI 0.595–1.000).^3^

**Table 3.** Strongest directed edges of temporal network from wave 1 to wave 2

Wave 1 to wave 2 measure	Directed edge weight *d*	Wave 1 to wave 2 measure	Directed edge weight *d*
Somatic symptoms→processing speed	−3.598	Interpersonal problems→age	−0.079
Depressed mood→face recognition	−0.997	Estradiol→depressed mood	−0.075
Anxiety symptoms→processing speed	−0.596	Depressed mood→somatic symptoms	−0.069
Menopausal status→processing speed	−0.391	Estradiol→interpersonal problems	−0.066
FSH→processing speed	−0.291	Somatic symptoms→age	−0.061
Age→processing speed	−0.285	Interpersonal problems→face recognition	−0.058
Somatic symptoms→working memory	−0.253	Positive affect→working memory	0.174
Positive affect→depressed mood	−0.186	Somatic symptoms→depressed mood	0.202
Positive affect→age	−0.173	Face recognition→processing speed	0.217
Positive affect→interpersonal problems	−0.160	Depressed mood→working memory	0.294
Working memory→face recognition	−0.156	Verbal memory→face recognition	0.467
Interpersonal problems→verbal memory	−0.153	Working memory→processing speed	0.540
Estradiol→FSH	−0.146	Positive affect→face recognition	0.558
Menopausal status→positive affect	−0.131	Estradiol→processing speed	0.793
Age→face recognition	−0.128	Interpersonal problems→processing speed	0.819
Depressed mood→verbal memory	−0.117	Somatic symptoms→face recognition	0.902
Somatic symptoms→positive affect	−0.107	Depressed mood→processing speed	1.016
Anxiety symptoms→working memory	−0.089	Positive affect→processing speed	9.583

FSH, follicle-stimulating hormone (mIU/mL); W1, wave 1; W2, wave 2.

## Discussion

The current study offers a novel network perspective on scar and vulnerability hypotheses which propose inverse cross-sectional and prospective links between cognitive functioning and depression and anxiety components. Overall, CLPN-derived contemporaneous and temporal networks across two time-points partially aligned with our study hypotheses, in which some but not all features of depression, anxiety, and cognitive functioning related to one another in the expected direction. To further our theoretical and applied understanding of this research question, we provide plausible accounts for the present findings.

Between clusters, why did greater interpersonal issues (e.g. feeling lonely, viewing others as unfriendly) and depressed mood, but not anxiety severity, correlate with reduced facial recognition? These results concur with and might be accounted for by evidence that depression (*v*. anxiety) symptoms and related facets had stronger associations with various deficits in social cognition (e.g. perspective-taking, affective and cognitive theory-of-mind) (Bora & Berk, 2016; Zainal & Newman, 2018). Lower processing speed was associated with more interpersonal problems and anxiety severity. Such findings extend the attentional control theory (Derakshan & Eysenck, 2009) by suggesting that anxiety features (e.g. worry, attentional biases toward threat) and relationship issues could consume finite attention and cognitive resources, thus correlating with a slower rate of processing (Nikolin et al., 2021; Zainal & Newman, 2021c). In addition, higher depressed mood and somatic symptoms (*v*. anxiety severity) coincided with lower verbal memory or WM. This pattern aligned with evidence that salient correlates of depression, but not anxiety, included compromised information tracking, retention, and recall abilities (Lyche, Jonassen, Stiles, Ulleberg, & Landrø, 2011; Zainal & Newman, 2021a). This could be because issues related to anhedonia and differentiating and registering negative (*v*. positive or neutral) material were more salient in depression (*v*. anxiety) (Dillon & Pizzagalli, 2018). Future studies should continue to examine the *specificity* of depression and anxiety components to unique cognitive functioning domains.

Interestingly, based on the two-step bridge EI values of contemporaneous networks, positive affect and anxiety severity (*v*. depressed mood, somatic symptoms, interpersonal problems) were markedly more likely to correspond with reduced cognitive functioning nodes. Findings might mean that positive affect-induced increased dopamine in frontal-subcortical reward-related brain networks is associated with enhanced attentional control, EF, and related information processing abilities (Yang, Yang, & Isen, 2013). The results could also be interpreted to be largely concordant with hypotheses arguing that excessive anxiety and related negative affect deplete finite frontoparietal-linked cognitive functioning resources, interfere with optimal engagement in the task-at-hand, and reduce motivation for cognitively stimulating activities (cf. attentional control theory and the *c*-factor of psychopathology; Abramovitch et al., 2021). Also, bridge EI analyses revealed that decreased WM and verbal memory showed the highest odds of activating nodes in depression and anxiety clusters cross-sectionally. This might be because abilities that capture executive functioning and verbal recall (*v*. processing speed and face recognition) capacities tend to be more coupled with intact activities of daily living, stable movement patterns, and lifestyle patterns (e.g. frequency, intensity, and duration of physical exercises) (Patience et al., 2019). Future prospective studies using network analysis can test the validity of these propositions.

Notably, the pattern of contemporaneous regularized partial network relations did not necessarily map on to temporal network relations. Another essential observation was that CLPN-derived results were consistent with the scar theory rather than the vulnerability theory. In other words, no cognitive functioning nodes were markedly associated with future depression and anxiety nodes. However, specific depression and anxiety nodes were connected to future unique cognitive functioning nodes. For example, reduced processing speed was related to previous higher somatic and anxiety severity (*v*. other depression nodes). Findings are congruous with the somatic marker hypothesis (Baddeley, 2013) that proposes vegetative-related depression and anxiety features (e.g. irritability, decreased effort, insomnia, appetite changes) could limit productive activities and negatively relate to future processing speed and WM domains. Also, we observed that lower WM correlated with previous greater levels of interpersonal problems, somatic symptoms, and anxiety severity. Based on some evidence (Baker, Kane, & Russell, 2020), issues of irritability, poor conflict resolution, communication skills deficits, and social withdrawal tendencies could, over time, worsen the ability to monitor information and make informed decisions in real-time optimally. Also, poorer face recognition was linked to prior greater depressed mood instead of other depression and anxiety nodes. This might be because prolonged depressed mood (*v*. other nodes) was more likely to persistently reduce exposure to faces (i.e. chances to identify emotions accurately) and adversely affect meta-memory and related meta-cognitive capacities (Turano & Viggiano, 2017). These reasons could also explain why compromised verbal memory was associated with previous higher levels of two depression nodes – depressed mood and interpersonal problems. Collectively, the current study outcomes are compatible with hypotheses (Nuno, Gomez-Benito, Carmona, & Pino, 2021) that elevated anxiety and depression correlate with cognitive functioning inefficiencies and recall deficits later. This process might occur via prolonged wear-and-tear of neurobiological brain areas entwined with social cognition, verbal memory, and executive functioning (e.g. inhibitory control, cognitive flexibility) (Zainal & Newman, 2022). Lifestyle factors (e.g. chronic sluggishness, fewer meaningful activities, lack of exercise), motivational deficits (e.g. anhedonia), and chronic tendency to experience diverse negative emotions might also be factors contributing to our current observations and merit the attention of future studies.

Relatedly, why was positive affect connected to better future face recognition, processing speed, and WM? Plausibly, based on prior prospective evidence (Johnson & Fredrickson, 2005), positive emotions facilitated increased socialization that could enhance accuracy of globally identifying, encoding, and recalling facial and emotional features and expressions. Furthermore, enhanced positive affect could be associated with improved WM and processing speed across time by raising mental flexibility (e.g. prompting thoughts of alternative perspectives and options) and participation in mentally stimulating tasks (Carpenter, Peters, Vastfjall, & Isen, 2013). Future longitudinal network investigations can evaluate how viable these conjectures are.

The present study had some limitations. First, single-item measures of cognitive functioning were used. Replication studies could minimize measurement error by using multiple assessments to represent cognitive functioning nodes in their network analyses. Second, the face recognition test had socio-cognitive and cultural biases (e.g. imbalanced representation of cultural groups in the stimuli set) (Less, 2012; Pearson Clinical Assessment, 2022) and was outdated. Future studies should use up-to-date tests that yield scores with more robust cross-cultural reliability and validity (e.g. Cambridge Face Memory Test; Duchaine & Nakayama, 2006; McKone et al., 2012). Additionally, future studies could test if the current study findings were replicated with established anxiety severity measures. Fourth, our CLPN approach could not separate between within-person and between-person variance. As between- and within-person effects may differ in magnitude and direction (Zainal & Newman, 2021a), future CLPN on mental health problems–cognitive functioning relations could use multilevel vector autoregressive models with intensive longitudinal designs (Epskamp, 2020). Fifth, given the all-female sample, the pattern of results might not generalize to males. Last, although CLPN empirically detected the non-zero estimated edges observed herein, it did not mean that these unique depression and anxiety components were related to cognitive impairment (e.g. dementia). It is possible that the degree of reduction in cognitive function domains was small. Relatedly, the cognitive function measures in the current study are infrequently, if at all, needed to engage in daily tasks in the real world. Therefore, future prospective network analysis should administer ecologically valid or mobile cognitive tests (e.g. Chinner, Blane, Lancaster, Hinds, & Koychev, 2018; Wolf, Dahl, Auen, & Doherty, 2017) to determine the extent to which findings extend to everyday cognitive functioning. Despite these shortcomings, the study's strengths include the large sample of community adult women who were diverse in terms of race, ethnicity, and education, its longitudinal design, and the use of a potent technique that offered more information than traditional statistics (e.g. ordinary least squares, SEM). In addition, all true edges that emerged in contemporaneous and temporal networks adjusted for the effects of other nodes and edges, baseline scores, and variables (age, menopausal status, estradiol, follicle-stimulating hormone). Moreover, our findings that later menopausal stages and higher levels of estradiol and follicle-stimulating hormone were related to lower processing speed replicates and extends previous studies (Hogervorst, Craig, & O'Donnell, 2021). It also highlights the importance of adjusting for menopausal status and possible hormone replacement therapy in future studies that recruit an all-women sample.

In closing, how can the current CLPN-derived findings translate to clinical practice? Since temporal networks showed positive affect had the largest relation to future nodes, enhancing positive emotions should be a key treatment target. Our findings highlight the importance of improving depression-linked relationship issues (e.g. perceived and objective social isolation) and ameliorating anxiety and somatic symptoms. Evidence-based interpersonal- and positive psychology-focused cognitive–behavioral therapies (Therond et al., 2021; Yates, Tyrell, & Masten, 2015) may thus be augmented by emphasizing the protective and enhancing effects of repeatedly practicing therapy skills on depression and anxiety symptoms *and* cognitive functioning. Such efforts can be delivered via face-to-face or telehealth therapies and digital mental health apps to prevent and treat anxiety, depression, and cognitive dysfunction (Ma et al., 2020). Furthermore, intensive values-driven pleasant activities scheduling, interpersonal effectiveness, and related therapy exercises may be more effective if paired with cognitive remediation (i.e. training persons to engage in cognitive-stimulating tasks to boost WM systematically). Simultaneously, because standalone cognitive remediation lacks far-transfer effects (Smid, Karbach, & Steinbeis, 2020), *augmentative* approaches may help those with or at-risk for depression and anxiety to stay mentally sharp in various contexts as they age (Mongia & Hechtman, 2012). Moreover, future augmentative efforts should include ecologically valid mobile cognitive functioning tests to evaluate its generalizability to daily life settings. Collectively, clinical science can profit from testing the efficacy of these innovative methods with well-powered gold-standard randomized controlled trials and dismantling studies.

## Footnotes

† The notes appear after the main text.

## Notes

1 The online Supplemental material details an exploratory factor analysis and confirmatory factor analysis of this scale.

2 The online Supplemental material details how data on menopausal status and biomarkers were defined and collected.

3 The online Supplemental material offers details on the relations among biomarker variables (FSH, estradiol, menopausal status) and primary nodes of interest (depression, anxiety, cognitive functioning).

## Supplementary material

For supplementary material accompanying this paper visit https://doi.org/10.1017/S0033291722000848. click here to view supplementary material

## References

[ref1] Abramovitch, A., Short, T., & Schweiger, A. (2021). The C factor: Cognitive dysfunction as a transdiagnostic dimension in psychopathology. Clinical Psychology Review, 86, 102007. doi: 10.1016/j.cpr.2021.102007. 33864968

[ref2] Albert, M., Smith, L. A., Scherr, P. A., Taylor, J. O., Evans, D. A., & Funkenstein, H. H. (1991). Use of brief cognitive tests to identify individuals in the community with clinically diagnosed Alzheimer's disease. International Journal of Neuroscience, 57, 167–178. doi: 10.3109/00207459109150691. 1938160

[ref3] Baddeley, A. (2013). Working memory and emotion: Ruminations on a theory of depression. Review of General Psychology, 17, 20–27. doi: 10.1037/a0030029.

[ref4] Baker, L. R., Kane, M. J., & Russell, V. M. (2020). Romantic partners’ working memory capacity facilitates relationship problem resolution through recollection of problem-relevant information. Journal of Experimental Psychology: General, 149, 580–584. doi: 10.1037/xge0000659. 31318255

[ref5] Barak, Y., Barson, D., Davie, G., Glue, P., & Paleacu, D. (2021). Internalize at your peril: Internalizing disorders as risk factors for dementia-cohort study. Geroscience, 43, 253–261. doi: 10.1007/s11357-020-00285-y. 33067707 PMC8050167

[ref6] Bates, M. E., & Lemay, E. P. (2004). The d2 test of attention: Construct validity and extensions in scoring techniques. Journal of the International Neuropsychological Society, 10, 392–400. doi: 10.1017/S135561770410307X. 15147597

[ref7] Berent-Spillson, A., Persad, C. C., Love, T., Sowers, M., Randolph, J. F., Zubieta, J. K., & Smith, Y. R. (2012). Hormonal environment affects cognition independent of age during the menopause transition. Journal of Clinical Endocrinology and Metabolism, 97, E1686–E1694. doi: 10.1210/jc.2012-1365. 22730514 PMC3431577

[ref8] Bernstein, E. E., Heeren, A., & McNally, R. J. (2017). Unpacking rumination and executive control: A network perspective. Clinical Psychological Science, 5, 816–826. doi: 10.1177/2167702617702717.

[ref9] Bernstein, E. E., Kleiman, E. M., van Bork, R., Moriarity, D. P., Mac Giollabhui, N., McNally, R. J., … Alloy, L. B. (2019). Unique and predictive relationships between components of cognitive vulnerability and symptoms of depression. Depression and Anxiety, 36, 950–959. doi: 10.1002/da.22935. 31332887 PMC6777955

[ref10] Bora, E., & Berk, M. (2016). Theory of mind in major depressive disorder: A meta-analysis. Journal of Affective Disorders, 191, 49–55. doi: 10.1016/j.jad.2015.11.023. 26655114

[ref11] Bromberger, J. T., Kravitz, H. M., Chang, Y., Randolph, J. F., Jr., Avis, N. E., Gold, E. B., & Matthews, K. A. (2013). Does risk for anxiety increase during the menopausal transition? Study of women's health across the nation. Menopause, 20, 488–495. doi: 10.1097/GME.0b013e3182730599. 23615639 PMC3641149

[ref12] Burcusa, S. L., & Iacono, W. G. (2007). Risk for recurrence in depression. Clinical Psychology Review, 27, 959–985. doi: 10.1016/j.cpr.2007.02.005. 17448579 PMC2169519

[ref13] Byrne, M. E., Tanofsky-Kraff, M., Lavender, J. M., Parker, M. N., Shank, L. M., Swanson, T. N., … Yanovski, J. A. (2021). Bridging executive function and disinhibited eating among youth: A network analysis. International Journal of Eating Disorders, 54, 721–732. doi: 10.1002/eat.23476. 33502799 PMC8119329

[ref14] Carpenter, S. M., Peters, E., Vastfjall, D., & Isen, A. M. (2013). Positive feelings facilitate working memory and complex decision making among older adults. Cognition and Emotion, 27, 184–192. doi: 10.1080/02699931.2012.698251. 22764739

[ref15] Chang, W. C., Wong, C. S. M., Or, P. C. F., Chu, A. O. K., Hui, C. L. M., Chan, S. K. W., … Chen, E. Y. H. (2020). Inter-relationships among psychopathology, premorbid adjustment, cognition and psychosocial functioning in first-episode psychosis: A network analysis approach. Psychological Medicine, 50, 2019–2027. doi: 10.1017/S0033291719002113. 31451127

[ref16] Chen, Y., Norton, D., McBain, R., Ongur, D., & Heckers, S. (2009). Visual and cognitive processing of face information in schizophrenia: Detection, discrimination and working memory. Schizophrenia Research, 107, 92–98. doi: 10.1016/j.schres.2008.09.010. 18947982 PMC2640943

[ref17] Chinner, A., Blane, J., Lancaster, C., Hinds, C., & Koychev, I. (2018). Digital technologies for the assessment of cognition: A clinical review. Evidence Based Mental Health, 21, 67. doi: 10.1136/eb-2018-102890. 29678927 PMC10270380

[ref18] Clayton, M. G., Giletta, M., Boettiger, C. A., & Prinstein, M. J. (2021). Determinants of excessive reassurance-seeking: Adolescents’ internalized distress, friendship conflict, and inhibitory control as prospective predictors. Journal of Clinical Child & Adolescent Psychology, 50, 88–96. doi: 10.1080/15374416.2019.1604234. 31050555 PMC6825879

[ref19] Cosco, T. D., Prina, M., Stubbs, B., & Wu, Y. T. (2017). Reliability and validity of the Center for Epidemiologic Studies Depression Scale in a population-based cohort of middle-aged U.S. adults. Journal of Nursing Measurement, 25, 476–485. doi: 10.1891/1061-3749.25.3.476. 29268830

[ref20] Derakshan, N., & Eysenck, M. W. (2009). Anxiety, processing efficiency, and cognitive performance. European Psychologist, 14, 168–176. doi: 10.1027/1016-9040.14.2.168.

[ref21] Dillon, D. G., & Pizzagalli, D. A. (2018). Mechanisms of memory disruption in depression. Trends in Neurosciences, 41, 137–149. doi: 10.1016/j.tins.2017.12.006. 29331265 PMC5835184

[ref22] Duchaine, B., & Nakayama, K. (2006). The Cambridge Face Memory Test: Results for neurologically intact individuals and an investigation of its validity using inverted face stimuli and prosopagnosic participants. Neuropsychologia, 44, 576–585. doi: 10.1016/j.neuropsychologia.2005.07.001. 16169565

[ref23] Eadeh, H. M., Markon, K. E., Nigg, J. T., & Nikolas, M. A. (2021). Evaluating the viability of neurocognition as a transdiagnostic construct using both latent variable models and network analysis. Research on Child and Adolescent Psychopathology, 49, 697–710. doi: 10.1007/s10802-021-00770-8. 33534092 PMC8713463

[ref24] Epskamp, S. (2020). Psychometric network models from time-series and panel data. Psychometrika, 85, 206–231. doi: 10.1007/s11336-020-09697-3. 32162233 PMC7186258

[ref25] Epskamp, S., Borsboom, D., & Fried, E. I. (2018). Estimating psychological networks and their accuracy: A tutorial paper. Behavior Research Methods, 50, 195–212. doi: 10.3758/s13428-017-0862-1. 28342071 PMC5809547

[ref26] Epskamp, S., Cramer, A. O., Waldorp, L. J., Schmittmann, V. D., & Borsboom, D. (2012). qgraph: Network visualizations of relationships in psychometric data. Journal of Statistical Software, 48, 1–18. doi: 10.18637/jss.v048.i04.

[ref27] Ettman, C. K., Abdalla, S. M., Cohen, G. H., Sampson, L., Vivier, P. M., & Galea, S. (2020). Prevalence of depression symptoms in US adults before and during the COVID-19 pandemic. JAMA Network Open, 3, e2019686. doi: 10.1001/jamanetworkopen.2020.19686. 32876685 PMC7489837

[ref28] Friedman, J., Hastie, T., & Tibshirani, R. (2010). Regularization paths for generalized linear models via coordinate descent. Journal of Statistical Software, 33, 1–22. https://www.jstatsoft.org/v33/i01/. 20808728 PMC2929880

[ref29] Galderisi, S., Rucci, P., Kirkpatrick, B., Mucci, A., Gibertoni, D., Rocca, P, … Italian Network for Research on Psychoses (2018). Interplay among psychopathologic variables, personal resources, context-related factors, and real-life functioning in individuals with schizophrenia: A network analysis. JAMA Psychiatry, 75, 396–404. doi: 10.1001/jamapsychiatry.2017.4607. 29450447 PMC5875306

[ref30] Galimberti, C., Bosi, M. F., Caricasole, V., Zanello, R., Dell'Osso, B., & Vigano, C. A. (2020). Using network analysis to explore cognitive domains in patients with unipolar versus bipolar depression: A prospective naturalistic study. CNS Spectrums, 25, 380–391. doi: 10.1017/S1092852919000968. 31060642

[ref31] Greendale, G. A., Wight, R. G., Huang, M. H., Avis, N., Gold, E. B., Joffe, H., … Karlamangla, A. S. (2010). Menopause-associated symptoms and cognitive performance: Results from the study of women's health across the nation. American Journal of Epidemiology, 171, 1214–1224. doi: 10.1093/aje/kwq067. 20442205 PMC2915492

[ref32] Haslbeck, J. M. B., & Waldorp, L. J. (2018). How well do network models predict observations? On the importance of predictability in network models. Behavior Research Methods, 50, 853–861. doi: 10.3758/s13428-017-0910-x. 28718088 PMC5880858

[ref33] Hogervorst, E., Craig, J., & O'Donnell, E. (2021). Cognition and mental health in menopause: A review. Best Practice & Research: Clinical Obstetrics & Gynaecology. 1-16. doi: 10.1016/j.bpobgyn.2021.10.009. 34969617

[ref34] Hoorelbeke, K., Marchetti, I., De Schryver, M., & Koster, E. H. (2016). The interplay between cognitive risk and resilience factors in remitted depression: A network analysis. Journal of Affective Disorders, 195, 96–104. doi: 10.1016/j.jad.2016.02.001 26878206

[ref35] Jia, Q. F., Yang, H. X., Zhuang, N. N., Yin, X. Y., Zhu, Z. H., Yuan, Y., … Hui, L. (2020). The role of lipoprotein profile in depression and cognitive performance: A network analysis. Scientific Reports, 10, 20704. doi: 10.1038/s41598-020-77782-9 33244178 PMC7693273

[ref36] Jiang, S. Y., Shan, H. D., Zhang, R. T., Lui, S. S. Y., Yang, H. X., Cheung, E. F. C., … Chan, R. C. K. (2022). Network analysis of executive function, emotion, and social anhedonia. PsyCh Journal, 11, 232–234. doi: 10.1002/pchj.444. 33783123

[ref37] Johnson, K. J., & Fredrickson, B. L. (2005). ‘We all look the same to me’: Positive emotions eliminate the own-race bias in face recognition. Psychological Science, 16, 875–881. doi: 10.1111/j.1467-9280.2005.01631.x. 16262774 PMC1808554

[ref38] Jones, P. (2020). networktools: Tools for identifying important nodes in networks. R package version 1.2.3. https://CRAN.R-project.org/package=networktools.

[ref39] Kravitz, H. M., Schott, L. L., Joffe, H., Cyranowski, J. M., & Bromberger, J. T. (2014). Do anxiety symptoms predict major depressive disorder in midlife women? The Study of Women's Health Across the Nation (SWAN) Mental Health Study (MHS). Psychological Medicine, 44, 2593–2602. doi: 10.1017/S0033291714000075. 24467997 PMC4135380

[ref40] Lee, T., & Shi, D. (2021). A comparison of full information maximum likelihood and multiple imputation in structural equation modeling with missing data. Psychological Methods, 26, 466–485. doi: 10.1037/met0000381. 33507765

[ref41] Less, A. D. (2012). Cultural biases in the Wechsler Memory Scale III *(*WMS-III*)*. UNF Graduate Theses and Dissertations, p. 591. https://digitalcommons.unf.edu/etd/591.

[ref42] Lyche, P., Jonassen, R., Stiles, T. C., Ulleberg, P., & Landrø, N. I. (2011). Verbal memory functions in unipolar major depression with and without co-morbid anxiety. The Clinical Neuropsychologist, 25, 359–375. doi: 10.1080/13854046.2010.547518. 21391152

[ref43] Ma, R., Mann, F., Wang, J., Lloyd-Evans, B., Terhune, J., Al-Shihabi, A., & Johnson, S. (2020). The effectiveness of interventions for reducing subjective and objective social isolation among people with mental health problems: A systematic review. Social Psychiatry and Psychiatric Epidemiology, 55, 839–876. doi: 10.1007/s00127-019-01800-z. 31741017 PMC7303071

[ref44] Madley-Dowd, P., Hughes, R., Tilling, K., & Heron, J. (2019). The proportion of missing data should not be used to guide decisions on multiple imputation. Journal of Clinical Epidemiology, 110, 63–73. doi: 10.1016/j.jclinepi.2019.02.016. 30878639 PMC6547017

[ref45] McKone, E., Stokes, S., Liu, J., Cohan, S., Fiorentini, C., Pidcock, M., … Pelleg, M. (2012). A robust method of measuring other-race and other-ethnicity effects: The Cambridge Face Memory Test format. PLoS ONE, 7, e47956. doi: 10.1371/journal.pone.0047956. 23118912 PMC3484147

[ref46] Mongia, M., & Hechtman, L. (2012). Cognitive behavior therapy for adults with attention-deficit/hyperactivity disorder: A review of recent randomized controlled trials. Current Psychiatry Reports, 14, 561–567. doi: 10.1007/s11920-012-0303-x. 22878974

[ref47] Newman, M. G., Jacobson, N. C., Zainal, N. H., Shin, K. E., Szkodny, L. E., & Sliwinski, M. J. (2019). The effects of worry in daily life: An ecological momentary assessment study supporting the tenets of the contrast avoidance model. Clinical Psychological Science, 7, 794–810. doi: 10.1177/2167702619827019. 31372313 PMC6675025

[ref48] Nikolin, S., Tan, Y. Y., Schwaab, A., Moffa, A., Loo, C. K., & Martin, D. (2021). An investigation of working memory deficits in depression using the n-back task: A systematic review and meta-analysis. Journal of Affective Disorders, 284, 1–8. doi: 10.1016/j.jad.2021.01.084. 33581489

[ref49] Nuno, L., Gomez-Benito, J., Carmona, V. R., & Pino, O. (2021). A systematic review of executive function and information processing speed in major depression disorder. Brain Sciences, 11, 147. doi: 10.3390/brainsci11020147. 33499360 PMC7912411

[ref50] Patience, J., Lai, K. S. P., Russell, E., Vasudev, A., Montero-Odasso, M., & Burhan, A. M. (2019). Relationship between mood, thinking, and walking: A systematic review examining depressive symptoms, executive function, and gait. American Journal of Geriatric Psychiatry, 27, 1375–1383. doi: 10.1016/j.jagp.2019.07.007. 31420232

[ref51] Pearson Clinical Assessment. (2022). WMS-III to WMS-IV: Rationale for change. Retrieved January 5, 2022, from http://images.pearsonclinical.com/images/products/wms-iv/wms-rationaleforchange.pdf.

[ref52] Pereira, D. R., Costa, P., & Cerqueira, J. J. (2015). Repeated assessment and practice effects of the written symbol digit modalities test using a short inter-test interval. Archives of Clinical Neuropsychology, 30, 424–434. doi: 10.1093/arclin/acv028. 25994156

[ref53] Psychological Corporation (1997). WAIS-III and WMS-III: Technical manual. San Antonio, TX: Psychological Corporation/Harcourt Brace.

[ref54] R Core Team. (2021). R: A language and environment for statistical computing. Vienna, Austria: R Foundation for Statistical Computing. https://www.R-project.org/.

[ref55] Romer, A. L., & Pizzagalli, D. A. (2021). Is executive dysfunction a risk marker or consequence of psychopathology? A test of executive function as a prospective predictor and outcome of general psychopathology in the adolescent brain cognitive development study. Developmental Cognitive Neuroscience, 51, 100994. doi: 10.1016/j.dcn.2021.100994. 34332330 PMC8340137

[ref56] Schmank, C. J., Goring, S. A., Kovacs, K., & Conway, A. R. A. (2019). Psychometric network analysis of the Hungarian WAIS. Journal of Intelligence, 7, 21–34. doi: 10.3390/jintelligence7030021. 31505834 PMC6789747

[ref57] Seelye, A. M., Howieson, D. B., Wild, K. V., Moore, M. M., & Kaye, J. A. (2009). Wechsler Memory Scale-III faces test performance in patients with mild cognitive impairment and mild Alzheimer's disease. Journal of Clinical and Experimental Neuropsychology, 31, 682–688. doi: 10.1080/13803390802484763. 19037811 PMC2829111

[ref58] Smid, C. R., Karbach, J., & Steinbeis, N. (2020). Toward a science of effective cognitive training. Current Directions in Psychological Science, 29, 531–537. doi: 10.1177/0963721420951599.

[ref59] Spitzer, R. L., Kroenke, K., Williams, J. W., & Löwe, B. (2006). A brief measure for assessing generalized anxiety disorder: The GAD-7. Archives of Internal Medicine, 166, 1092–1097. doi: 10.1001/archinte.166.10.1092. 16717171

[ref60] Therond, A., Pezzoli, P., Abbas, M., Howard, A., Bowie, C. R., & Guimond, S. (2021). The efficacy of cognitive remediation in depression: A systematic literature review and meta-analysis. Journal of Affective Disorders, 284, 238–246. doi: 10.1016/j.jad.2021.02.009. 33631438

[ref61] Turano, M. T., & Viggiano, M. P. (2017). The relationship between face recognition ability and socioemotional functioning throughout adulthood. Neuropsychology, Development, and Cognition. Section B, Aging, Neuropsychology and Cognition, 24, 613–630. doi: 10.1080/13825585.2016.1244247. 27754777

[ref62] van Bork, R., Rhemtulla, M., Waldorp, L. J., Kruis, J., Rezvanifar, S., & Borsboom, D. (2021). Latent variable models and networks: Statistical equivalence and testability. Multivariate Behavioral Research, 56, 175–198. doi: 10.1080/00273171.2019.1672515. 31617420

[ref63] van Buuren, S., & Groothuis-Oudshoorn, K. (2011). Mice: Multivariate imputation by chained equations in R. Journal of Statistical Software, 45, 1548–7660. doi: 10.18637/jss.v045.i03.

[ref64] van Wanrooij, L. L., Borsboom, D., Moll van Charante, E. P., Richard, E., & van Gool, W. A. (2019). A network approach on the relation between apathy and depression symptoms with dementia and functional disability. International Psychogeriatrics, 31, 1655–1663. doi: 10.1017/S1041610218002387. 30782219

[ref65] Wechsler, D. (1997). Wechsler Memory Scale-III. San Antonio, TX: The Psychological Corporation.

[ref66] Wolf, T. J., Dahl, A., Auen, C., & Doherty, M. (2017). The reliability and validity of the Complex Task Performance Assessment: A performance-based assessment of executive function. Neuropsychological Rehabilitation, 27, 707–721. doi: 10.1080/09602011.2015.1037771. 25939359 PMC4635076

[ref67] Wolters, F. J., Chibnik, L. B., Waziry, R., Anderson, R., Berr, C., Beiser, A., … Hofman, A. (2020). Twenty-seven-year time trends in dementia incidence in Europe and the United States: The Alzheimer Cohorts Consortium. Neurology, 95, e519–e531. doi: 10.1212/WNL.0000000000010022. 32611641 PMC7455342

[ref68] Yang, H., Yang, S., & Isen, A. M. (2013). Positive affect improves working memory: Implications for controlled cognitive processing. Cognition and Emotion, 27, 474–482. doi: 10.1080/02699931.2012.713325. 22917664

[ref69] Yates, T. M., Tyrell, F. A., & Masten, A. S. (2015). Resilience theory and the practice of positive psychology from individuals to societies. Positive Psychology in Practice, 773–788. doi: 10.1002/9781118996874.ch44.

[ref70] Zainal, N. H., & Newman, M. G. (2018). Worry amplifies theory-of-mind reasoning for negatively valenced social stimuli in generalized anxiety disorder. Journal of Affective Disorders, 227, 824–833. doi: 10.1016/j.jad.2017.11.084. 29254067 PMC6707505

[ref71] Zainal, N. H., & Newman, M. G. (2021a). Depression and executive functioning bidirectionally impair one another across 9 years: Evidence from within-person latent change and cross-lagged models. European Psychiatry, 64, e43. doi: 10.1192/j.eurpsy.2021.2217. 34134796 PMC8278253

[ref72] Zainal, N. H., & Newman, M. G. (2021b). Elevated anxious and depressed mood relate to future executive dysfunction in older adults: A longitudinal network analysis of psychopathology and cognitive functioning. *PsyArXiv.* doi:10.31234/osf.io/hrfqa. PMC10046395 36993876

[ref73] Zainal, N. H., & Newman, M. G. (2021c). Within-person increase in pathological worry predicts future depletion of unique executive functioning domains. Psychological Medicine, 51, 1676–1686. doi: 10.1017/S0033291720000422. 32188519 PMC7501084

[ref74] Zainal, N. H., & Newman, M. G. (2022). Inflammation mediates depression and generalized anxiety symptoms predicting executive function impairment after 18 years. Journal of Affective Disorders, 296, 465–475. doi: 10.1016/j.jad.2021.08.077. 34649180 PMC8603378

