# A cross-lagged prospective network analysis of depression and anxiety and cognitive functioning components in midlife community adult women – CORRIGENDUM

**DOI:** 10.1017/S0033291723003574

**Published:** 2023-12-01

**Authors:** Nur Hani Zainal, Michelle G. Newman

The Epskamp (2020) citation should be replaced with the Wysocki et al. (2022) citation.
