# Supplementary material for: A cross-lagged prospective network analysis of depression and anxiety and cognitive functioning components in midlife community adult women
Source: Psychol Med. 2022 May 10;53(9):4160–71. doi: 10.1017/S0033291722000848 (PMC9646930; doi:10.1017/S0033291722000848)
Supplement: Supplementary file 1 [file S0033291722000848sup001.docx]

**Supplemental Materials**

**Psychometric Properties of 4-Item Anxiety Measure**

**Exploratory Factor Analysis**

Prior to conducting the EFA, we determined that the Kaiser-Meyer-Olkin (KMO) test of sampling adequacy (Wave 1: 0.79) indicated that there was enough proportion of common variance among the items for EFA (Costello & Osborne, 2005). Furthermore, there was adequate covariances among the items for EFA, as indicated by the significant Bartlett’s test of sphericity (Wave 1: χ^2^(*df* = 60) = 6670.00, *p* < .001; Wave 2: χ^2^(*df* = 60) = 6670.00, *p* < .001). Moreover, the cases to item ratio (3302:4) with the original sample was sufficient. Pearson’s correlations among items were high (Wave 1: *r* = 0.543 to 0.758; Wave 2: *r* = 0.517 to 0.730). The number of factors to extract was determined via a scree plot and parallel analysis using optimized implementation on 1,000 correlation matrices in which eigenvalues at or above the 95^th^ percentile were regarded as critical values (Timmerman & Lorenzo-Seva, 2011). Since parallel analysis showed that the first component in the principal component analysis had eigenvalues larger than their parallel components, only one factor was retained. The one factor accounted for 66.26% of the total variance at Wave 1, and 62.61% at Wave 2. Item communalities were high at Wave 1 (0.574 to 0.772) and Wave 2 (0.507 to 0.755), whereas item uniqueness values were low at Wave 1 (0.228 to 0.426) and Wave 2 (0.245 to 0.493). Likewise, item loadings were high at Wave 1 (0.758 to 0.879) and Wave 2 (0.712 to 0.869).

**Confirmatory Factor Analysis**

CFA showed that the 4-item anxiety scale had good model fit at Wave 1 (χ^2^(*df* = 2) = 27.183, *p* < .001, confirmatory factor index (CFI) = .992, root mean square of approximation (RMSEA) = .062, 95% confidence interval (CI) = [.042–.083] and Wave 2 (χ^2^(*df* = 2) = 14.024, *p* = .001, CFI = .995, RMSEA = .043 [.024, .065]). Tables S2 and S3 presents the details of the CFAs. All standardized factor loadings were large and statistically significant. Likewise, all item intercepts and residual variances were statistically significant.

**Measure of Menopausal Status**

Menopausal status was defined using the following four self-reported categories (Luetters et al., 2007): (1) Premenopausal: Presence of highly predictable menstrual cycles between two waves of assessment; (2) Early perimenopause: Reduced predictability of menstrual cycle in the past 3 months between two waves of assessment; (3) Late perimenopause: Cessation of menses for 3 to 11 months; (4) Postmenopausal: No menses for 12 months or more.

**Procedures for Biomarker Data Collection**

Levels of estradiol (pg/mL) and follicle-stimulating hormone (mIU/mL) were collected via blood draw after participants fasted overnight, and occurred between Days 2 and 5 for menstruating women (Luetters et al., 2007). These bioassays were refrigerated 1-2 hours after venipuncture, and aliquotted, frozen, and shipped in batches to a laboratory where blood serum assays were uninterruptedly measured for estradiol and follicle-stimulating hormone with the ACS-180 automated analyzer (Bayer Diagnostics Corporation, Tarrytown, NY). Mean inter- and intra-assay coefficients of variation (CV) was 6.4% for estradiol, and 10.6% for follicle-stimulating hormone (Randolph et al., 2004).

**Relations Among Biomarker Variables and Network Nodes of Interest**

**Contemporaneous Networks**

Later stages of menopause were associated with lower positive affect (r = -0.086) and verbal memory (*r* = -0.030). Moreover, later menopausal stages were related to more depressed mood (*r* = 0.038) and interpersonal problems (*r* = 0.039). Also, higher estradiol level was connected with more positive affect (r = 0.048). Further, higher follicle-stimulating hormone level was related to reduced positive affect (*r* = -0.049) and processing speed (*r* = -0.020).

**Temporal Networks**

Later stages of menopause at W1 were related to lower W2 processing speed (d = -0.391) and positive affect (*d* = -0.131). In addition, higher W1 estradiol level was linked to lower W2 depressed mood (*d* = -0.075) and interpersonal problems (*d* = -0.066), and stronger W2 processing speed (*d* = 0.793). Simultaneously, greater W1 follicle-stimulating hormone level was associated with reduced W2 processing speed (*d* = -0.291).

Table S1

*Demographic and Related Study Variables at Wave 1 and Wave 2*

|  | Wave 1 | | | | | |  | Wave 2 | | | | | |
| --- | --- | --- | --- | --- | --- | --- | --- | --- | --- | --- | --- | --- | --- |
|  | *M/n* | *(SD/%)* | Min | Max | Skew | Kurt |  | *M/n* | *(SD/%)* | Min | Max | Skew | Kurt |
| Age | 53.281 | (53.281) | 49 | 61 | 0.711 | -0.228 |  | 54.813 | (2.872) | 50 | 62 | 0.705 | -0.079 |
| Estradiol (pg/mL) (Log) | 2.973 | (0.817) | 1.6 | 7.1 | 1.952 | 3.218 |  | 3.219 | (0.663) | 1.4 | 6.6 | 1.634 | 4.17 |
| FSH (mIU/mL) (Log) | -0.045 | (1.068) | -1.5 | 2.5 | 1.104 | 0.937 |  | 0.17 | (1.114) | -1.1 | 2.2 | 0.929 | -0.588 |
| Menopausal Status |  |  |  |  |  |  |  |  |  |  |  |  |  |
| Pre-Menopausal | 211 | (11.6) |  |  |  |  |  | 192 | (10.6) |  |  |  |  |
| Early Peri-Menopausal | 405 | (22.3) |  |  |  |  |  | 286 | (15.7) |  |  |  |  |
| Late Peri-Menopausal | 28 | (1.54) |  |  |  |  |  | 15 | (0.8) |  |  |  |  |
| Post-Menopausal | 1172 | (64.5) |  |  |  |  |  | 1323 | (72.9) |  |  |  |  |
| Race |  |  |  |  |  |  |  |  |  |  |  |  |  |
| Asian American | 242 | (13.3) |  |  |  |  |  |  |  |  |  |  |  |
| Black/African American | 482 | (26.5) |  |  |  |  |  |  |  |  |  |  |  |
| Another race | 285 | (15.7) |  |  |  |  |  |  |  |  |  |  |  |
| White American | 807 | (44.4) |  |  |  |  |  |  |  |  |  |  |  |
| Ethnicity |  |  |  |  |  |  |  |  |  |  |  |  |  |
| Hispanic or Latinx | 285 | (15.7) |  |  |  |  |  |  |  |  |  |  |  |
| Not Hispanic or Latinx | 1531 | (84.3) |  |  |  |  |  |  |  |  |  |  |  |
| Level of Formal Education |  |  |  |  |  |  |  |  |  |  |  |  |  |
| Less than High School | 198 | (10.9) |  |  |  |  |  |  |  |  |  |  |  |
| High school | 320 | (17.6) |  |  |  |  |  |  |  |  |  |  |  |
| Some college | 556 | (30.6) |  |  |  |  |  |  |  |  |  |  |  |
| College | 367 | (20.2) |  |  |  |  |  |  |  |  |  |  |  |
| Post-graduate education | 375 | (20.6) |  |  |  |  |  |  |  |  |  |  |  |
|  |  |  |  |  |  |  |  |  |  |  |  |  |  |

*Note.* FSH = follicle-stimulating hormone (mIU/mL).

Table S2

*CFA for Wave 1 Four-Item Anxiety Scale*

|  |  |  |  | 95% CI | Standardized |
| --- | --- | --- | --- | --- | --- |
|  | Estimate | (*SE*) | *p* | [Lower, Upper] | Estimate |
| Factor Loadings |  |  |  |  |  |
| Irritability | 1.000 | (0.000) | – | – | 0.799 |
| Nervous | 1.147 | (0.022) | 0.000 | [1.105, 1.19] | 0.866 |
| Accelerated HR | 0.778 | (0.026) | 0.000 | [0.728, 0.829] | 0.722 |
| Fearfulness | 0.935 | (0.025) | 0.000 | [0.887, 0.984] | 0.845 |
| Residual Variances |  |  |  |  |  |
| Irritability | 0.585 | (0.029) | 0.000 | [0.528, 0.642] | 0.362 |
| Nervous | 0.453 | (0.025) | 0.000 | [0.404, 0.501] | 0.250 |
| Accelerated HR | 0.574 | (0.023) | 0.000 | [0.528, 0.619] | 0.479 |
| Fearfulness | 0.360 | (0.018) | 0.000 | [0.325, 0.395] | 0.286 |
| Latent Anxiety | 1.030 |  | 0.000 | [0.943, 1.117] | 1.000 |
| Intercepts |  |  |  |  |  |
| Irritability | 2.167 | (0.022) | 0.000 | [2.124, 2.211] | 1.705 |
| Nervous | 2.218 | (0.023) | 0.000 | [2.172, 2.264] | 1.649 |
| Accelerated HR | 1.585 | (0.019) | 0.000 | [1.548, 1.622] | 1.448 |
| Fearfulness | 1.538 | (0.020) | 0.000 | [1.499, 1.576] | 1.369 |
| Latent Anxiety | 0.000 | (0.000) | – | – | – |
|  |  |  |  |  |  |

*Note.* CFA = confirmatory factor analysis; CI = confidence interval; HR = heart rate; SE = standard error.

Table S3

*CFA for Wave 2 Four-Item Anxiety Scale*

|  |  |  |  | 95% CI | Standardized |
| --- | --- | --- | --- | --- | --- |
|  | Estimate | (*SE*) | *p* | [Lower, Upper] | Estimate |
| Factor Loadings |  |  |  |  |  |
| Irritability | 1.000 | (0.000) | – | – | 0.726 |
| Nervous | 1.259 | (0.034) | 0.000 | [1.193, 1.325] | 0.820 |
| Accelerated HR | 0.931 | (0.032) | 0.000 | [0.868, 0.993] | 0.749 |
| Fearfulness | 1.109 | (0.034) | 0.000 | [1.041, 1.176] | 0.853 |
| Residual Variances |  |  |  |  |  |
| Irritability | 0.618 | (0.028) | 0.000 | [0.562, 0.674] | 0.472 |
| Nervous | 0.533 | (0.03) | 0.000 | [0.474, 0.592] | 0.327 |
| Accelerated HR | 0.470 | (0.022) | 0.000 | [0.427, 0.512] | 0.440 |
| Fearfulness | 0.318 | (0.018) | 0.000 | [0.282, 0.354] | 0.273 |
| Latent Anxiety | 0.691 | (0.041) | 0.000 | [0.611, 0.770] | 1.000 |
| Intercepts |  |  |  |  |  |
| Irritability | 2.011 | (0.02) | 0.000 | [1.972, 2.050] | 1.757 |
| Nervous | 2.117 | (0.022) | 0.000 | [2.074, 2.161] | 1.660 |
| Accelerated HR | 1.571 | (0.018) | 0.000 | [1.535, 1.606] | 1.520 |
| Fearfulness | 1.516 | (0.019) | 0.000 | [1.479, 1.553] | 1.403 |
| Latent Anxiety | 0.000 | (0.000) | – | – | – |
|  |  |  |  |  |  |

*Note.* CFA = confirmatory factor analysis; CI = confidence interval; HR = heart rate; SE = standard error.

Figure S1

*Two-Step Bridge Expected Influence for Contemporaneous Networks*

*Note.* anx = anxiety symptoms; dep = depressed mood; frg = face recognition; vrm = verbal memory; int = interpersonal problems; pa = positive affect; ps = processing speed; som = somatic symptoms; fsh = follicle-stimulating hormone (mIU/mL); est = estradiol (pg/mL); age = age of participants at respective wave; mns = menopausal status (pre-menopausal, early perimenopausal, late perimenopausal, and post-menopausal).

Figure S2

*Autoregression Coefficients of Cognitive Functioning and Mental Health Components*

*Note.* anx = anxiety symptoms; dep = depressed mood; frg = face recognition; vrm = verbal memory; int = interpersonal problems; pa = positive affect; ps = processing speed; som = somatic symptoms; fsh = follicle-stimulating hormone (mIU/mL); est = estradiol (pg/mL); age = age of participants at respective wave; mns = menopausal status (pre-menopausal, early perimenopausal, late perimenopausal, and post-menopausal).

References

Costello, A. B., & Osborne, J. (2005). Best practices in exploratory factor analysis: Four recommendations for getting the most from your analysis. *Practical Assessment, Research, and Evaluation, 10*, 1-10. doi:10.7275/jyj1-4868

Luetters, C., Huang, M. H., Seeman, T., Buckwalter, G., Meyer, P. M., Avis, N. E., . . . Greendale, G. A. (2007). Menopause transition stage and endogenous estradiol and follicle-stimulating hormone levels are not related to cognitive performance: cross-sectional results from the study of women's health across the nation (SWAN). *Journal of Women's Health, 16*, 331-344. doi:10.1089/jwh.2006.0057

Randolph, J. F., Jr., Sowers, M., Bondarenko, I. V., Harlow, S. D., Luborsky, J. L., & Little, R. J. (2004). Change in estradiol and follicle-stimulating hormone across the early menopausal transition: Effects of ethnicity and age. *Journal of Clinical Endocrinology and Metabolism, 89*, 1555-1561. doi:10.1210/jc.2003-031183

Timmerman, M. E., & Lorenzo-Seva, U. (2011). Dimensionality assessment of ordered polytomous items with parallel analysis. *Psychological Methods, 16*, 209-220. doi:10.1037/a0023353
