# Supplementary material for: A cross-lagged prospective network analysis of depression and anxiety and cognitive functioning components in midlife community adult women
Source: Psychol Med. Author manuscript; Available in PMC 2023 Sep 22. (PMC9646930; doi:10.1017/S0033291722000848)
Supplement: Supplementary file 1 [file NIHMS1816487-supplement-supplementary_material.pdf]

Supplemental Materials

Psychometric Properties of 4-Item Anxiety Measure

Exploratory Factor Analysis

Prior to conducting the EFA, we determined that the Kaiser-Meyer-Olkin (KMO) test of sampling adequacy (Wave 1: 0.79) indicated that there was enough proportion of common variance among the items for EFA (Costello & Osborne, 2005). Furthermore, there was adequate covariances among the items for EFA, as indicated by the significant Bartlett's test of sphericity (Wave 1: $\chi^2(df = 60) = 6670.00$, $p < .001$; Wave 2: $\chi^2(df = 60) = 6670.00$, $p < .001$). Moreover, the cases to item ratio (3302:4) with the original sample was sufficient. Pearson's correlations among items were high (Wave 1: $r = 0.543$ to 0.758; Wave 2: $r = 0.517$ to 0.730). The number of factors to extract was determined via a scree plot and parallel analysis using optimized implementation on 1,000 correlation matrices in which eigenvalues at or above the 95th percentile were regarded as critical values (Timmerman & Lorenzo-Seva, 2011). Since parallel analysis showed that the first component in the principal component analysis had eigenvalues larger than their parallel components, only one factor was retained. The one factor accounted for 66.26% of the total variance at Wave 1, and 62.61% at Wave 2. Item communalities were high at Wave 1 (0.574 to 0.772) and Wave 2 (0.507 to 0.755), whereas item uniqueness values were low at Wave 1 (0.228 to 0.426) and Wave 2 (0.245 to 0.493). Likewise, item loadings were high at Wave 1 (0.758 to 0.879) and Wave 2 (0.712 to 0.869).

Confirmatory Factor Analysis

CFA showed that the 4-item anxiety scale had good model fit at Wave 1 ($\chi^2(df = 2) = 27.183$, $p < .001$, confirmatory factor index (CFI) = .992, root mean square of approximation (RMSEA) = .062, 95% confidence interval (CI) = [.042–.083] and Wave 2 ($\chi^2(df = 2) = 14.024$, $p = .001$, CFI = .995, RMSEA = .043 [.024, .065]). Tables S2 and S3 presents the details of the CFAs. All standardized factor loadings were large and statistically significant. Likewise, all item intercepts and residual variances were statistically significant.

Measure of Menopausal Status

Menopausal status was defined using the following four self-reported categories (Luetters et al., 2007): (1) Premenopausal: Presence of highly predictable menstrual cycles between two waves of assessment; (2) Early perimenopause: Reduced predictability of menstrual cycle in the past 3 months between two waves of assessment; (3) Late perimenopause: Cessation of menses for 3 to 11 months; (4) Postmenopausal: No menses for 12 months or more.

Procedures for Biomarker Data Collection

Levels of estradiol (pg/mL) and follicle-stimulating hormone (mIU/mL) were collected via blood draw after participants fasted overnight, and occurred between Days 2 and 5 for menstruating women (Luetters et al., 2007). These bioassays were refrigerated 1-2 hours after venipuncture, and aliquotted, frozen, and shipped in batches to a laboratory where blood serum assays were uninterruptedly measured for estradiol and follicle-stimulating hormone with the ACS-180 automated analyzer (Bayer Diagnostics Corporation, Tarrytown, NY). Mean inter- and intra-assay coefficients of variation (CV) was 6.4% for estradiol, and 10.6% for follicle-stimulating hormone (Randolph et al., 2004).

Relations Among Biomarker Variables and Network Nodes of Interest

Contemporaneous Networks

Later stages of menopause were associated with lower positive affect (r = -0.086) and verbal memory (*r* = -0.030). Moreover, later menopausal stages were related to more depressed mood (*r* = 0.038) and interpersonal problems (*r* = 0.039). Also, higher estradiol level was connected with more positive affect (r = 0.048). Further, higher follicle-stimulating hormone level was related to reduced positive affect (*r* = -0.049) and processing speed (*r* = -0.020).

Temporal Networks

Later stages of menopause at W1 were related to lower W2 processing speed (d = -0.391) and positive affect (*d* = -0.131). In addition, higher W1 estradiol level was linked to lower W2 depressed mood (*d* = -0.075) and interpersonal problems (*d* = -0.066), and stronger W2 processing speed (*d* = 0.793). Simultaneously, greater W1 follicle-stimulating hormone level was associated with reduced W2 processing speed (*d* = -0.291).

Table S1

Demographic and Related Study Variables at Wave 1 and Wave 2

	Wave 1						Wave 2					
	M/n	*(SD/%)*	Min	Max	Skew	Kurt	*M/n*	*(SD/%)*	Min	Max	Skew	Kurt
Age	53.281	(53.281)	49	61	0.711	-0.228	54.813	(2.872)	50	62	0.705	-0.079
Estradiol (pg/mL) (Log)	2.973	(0.817)	1.6	7.1	1.952	3.218	3.219	(0.663)	1.4	6.6	1.634	4.17
FSH (mIU/mL) (Log)	-0.045	(1.068)	-1.5	2.5	1.104	0.937	0.17	(1.114)	-1.1	2.2	0.929	-0.588
Menopausal Status												
Pre-Menopausal	211	(11.6)					192	(10.6)				
Early Peri-Menopausal	405	(22.3)					286	(15.7)				
Late Peri-Menopausal	28	(1.54)					15	(0.8)				
Post-Menopausal	1172	(64.5)					1323	(72.9)				
Race												
Asian American	242	(13.3)										
Black/African American	482	(26.5)										
Another race	285	(15.7)										
White American	807	(44.4)										
Ethnicity												
Hispanic or Latinx	285	(15.7)										
Not Hispanic or Latinx	1531	(84.3)										
Level of Formal Education												
Less than High School	198	(10.9)										
High school	320	(17.6)										
Some college	556	(30.6)										
College	367	(20.2)										
Post-graduate education	375	(20.6)										

Note. FSH = follicle-stimulating hormone (mIU/mL).

Table S2

CFA for Wave 1 Four-Item Anxiety Scale

	Estimate	(*SE*)	*p*	95% CI [Lower, Upper]	Standardized Estimate
Factor Loadings					
Irritability	1.000	(0.000)	–	–	0.799
Nervous	1.147	(0.022)	0.000	[1.105, 1.19]	0.866
Accelerated HR	0.778	(0.026)	0.000	[0.728, 0.829]	0.722
Fearfulness	0.935	(0.025)	0.000	[0.887, 0.984]	0.845
Residual Variances					
Irritability	0.585	(0.029)	0.000	[0.528, 0.642]	0.362
Nervous	0.453	(0.025)	0.000	[0.404, 0.501]	0.250
Accelerated HR	0.574	(0.023)	0.000	[0.528, 0.619]	0.479
Fearfulness	0.360	(0.018)	0.000	[0.325, 0.395]	0.286
Latent Anxiety	1.030		0.000	[0.943, 1.117]	1.000
Intercepts					
Irritability	2.167	(0.022)	0.000	[2.124, 2.211]	1.705
Nervous	2.218	(0.023)	0.000	[2.172, 2.264]	1.649
Accelerated HR	1.585	(0.019)	0.000	[1.548, 1.622]	1.448
Fearfulness	1.538	(0.020)	0.000	[1.499, 1.576]	1.369
Latent Anxiety	0.000	(0.000)	–	–	–

Note. CFA = confirmatory factor analysis; CI = confidence interval; HR = heart rate; SE = standard error.

Table S3

CFA for Wave 2 Four-Item Anxiety Scale

	Estimate	(*SE*)	*p*	95% CI [Lower, Upper]	Standardized Estimate
Factor Loadings					
Irritability	1.000	(0.000)	–	–	0.726
Nervous	1.259	(0.034)	0.000	[1.193, 1.325]	0.820
Accelerated HR	0.931	(0.032)	0.000	[0.868, 0.993]	0.749
Fearfulness	1.109	(0.034)	0.000	[1.041, 1.176]	0.853
Residual Variances					
Irritability	0.618	(0.028)	0.000	[0.562, 0.674]	0.472
Nervous	0.533	(0.03)	0.000	[0.474, 0.592]	0.327
Accelerated HR	0.470	(0.022)	0.000	[0.427, 0.512]	0.440
Fearfulness	0.318	(0.018)	0.000	[0.282, 0.354]	0.273
Latent Anxiety	0.691	(0.041)	0.000	[0.611, 0.770]	1.000
Intercepts					
Irritability	2.011	(0.02)	0.000	[1.972, 2.050]	1.757
Nervous	2.117	(0.022)	0.000	[2.074, 2.161]	1.660
Accelerated HR	1.571	(0.018)	0.000	[1.535, 1.606]	1.520
Fearfulness	1.516	(0.019)	0.000	[1.479, 1.553]	1.403
Latent Anxiety	0.000	(0.000)	–	–	–

Note. CFA = confirmatory factor analysis; CI = confidence interval; HR = heart rate; SE = standard error.

Figure S1

Two-Step Bridge Expected Influence for Contemporaneous Networks

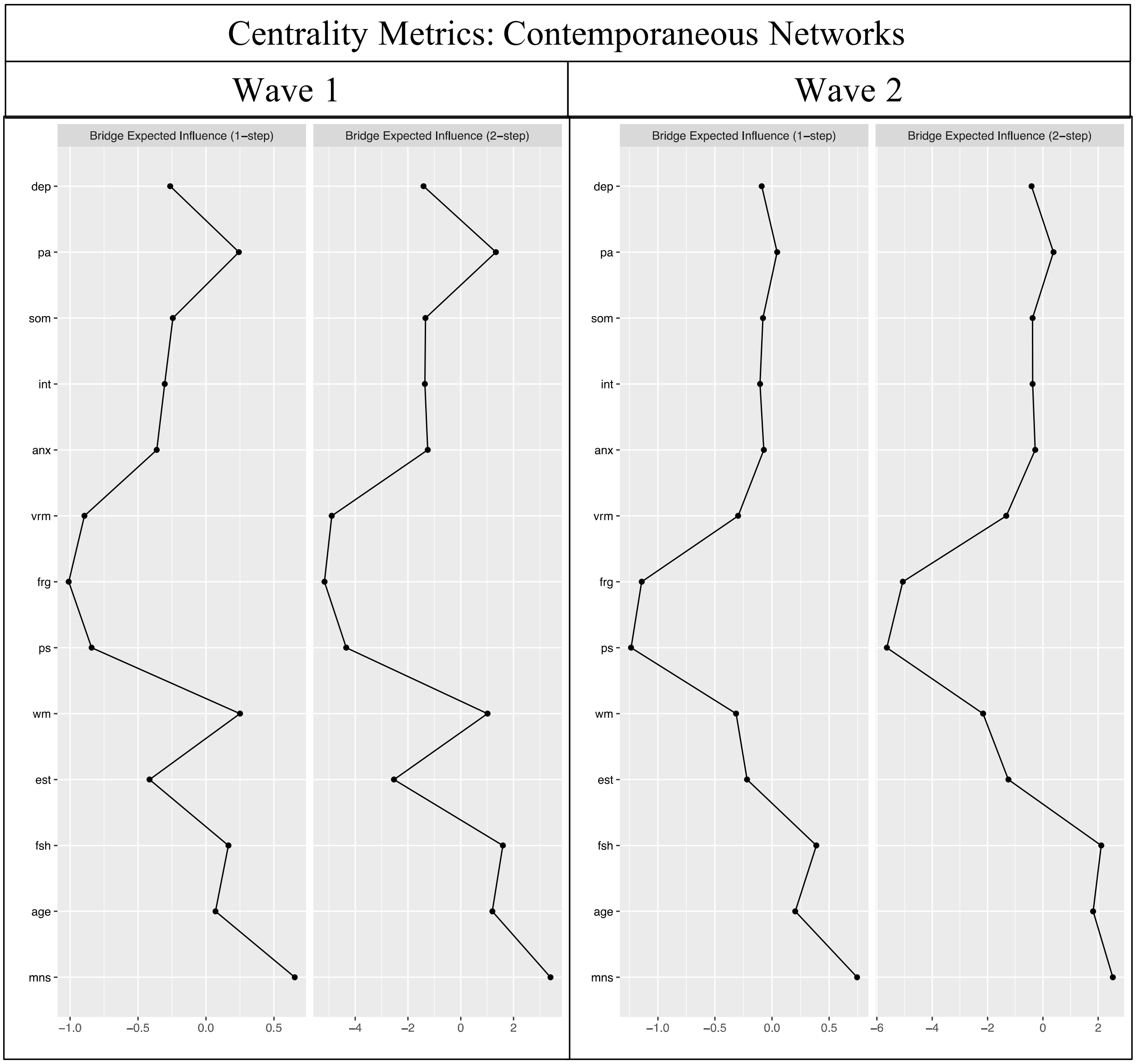

Note. anx = anxiety symptoms; dep = depressed mood; frg = face recognition; vrm = verbal memory; int = interpersonal problems; pa = positive affect; ps = processing speed; som = somatic symptoms; fsh = follicle-stimulating hormone (mIU/mL); est = estradiol (pg/mL); age = age of participants at respective wave; mns = menopausal status (pre-menopausal, early perimenopausal, late perimenopausal, and post-menopausal).

Figure S2

Autoregression Coefficients of Cognitive Functioning and Mental Health Components

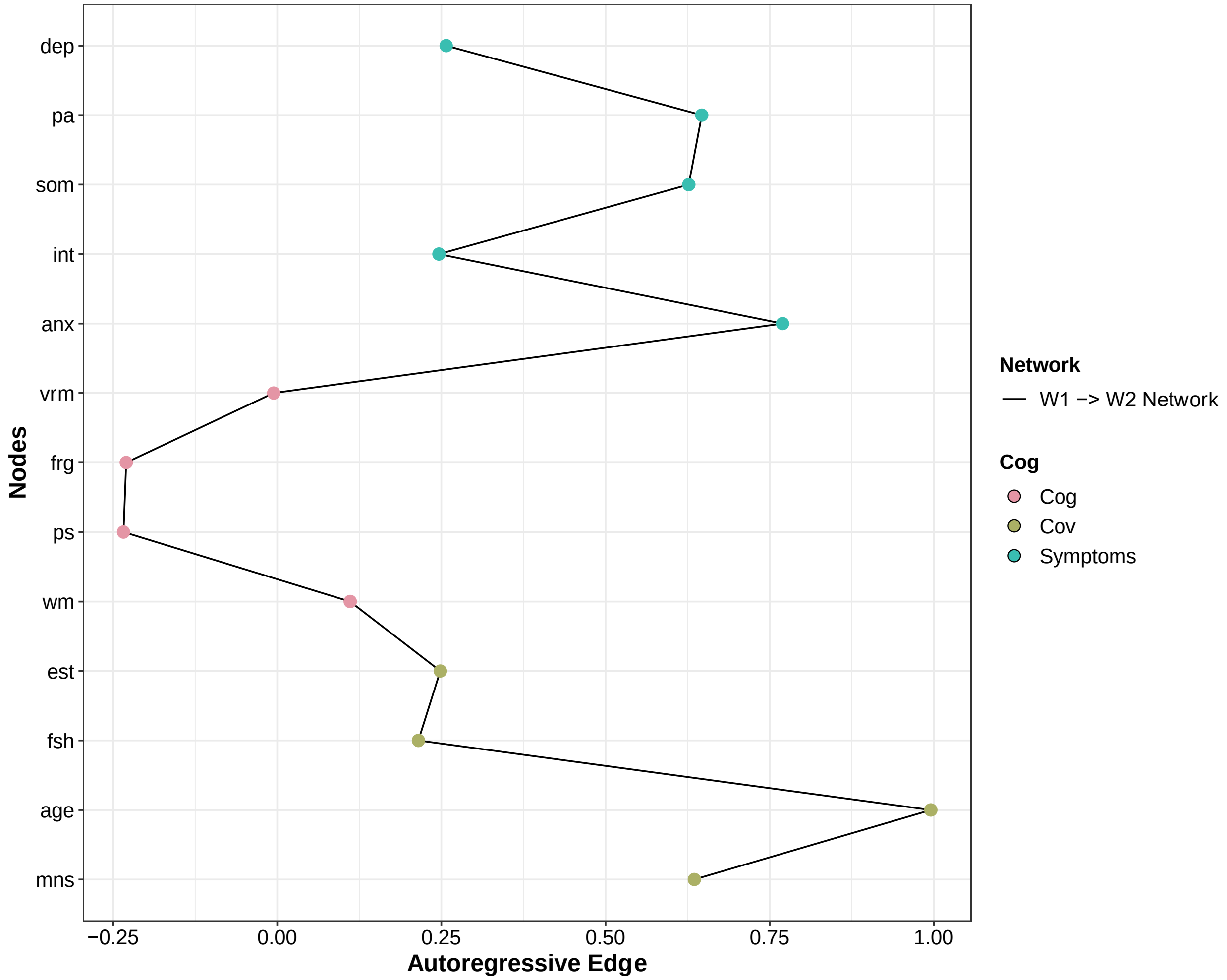

Note. anx = anxiety symptoms; dep = depressed mood; frg = face recognition; vrm = verbal memory; int = interpersonal problems; pa = positive affect; ps = processing speed; som = somatic symptoms; fsh = follicle-stimulating hormone (mIU/mL); est = estradiol (pg/mL); age = age of participants at respective wave; mns = menopausal status (pre-menopausal, early perimenopausal, late perimenopausal, and post-menopausal).

References

Costello, A. B., & Osborne, J. (2005). Best practices in exploratory factor analysis: Four recommendations for getting the most from your analysis. *Practical Assessment, Research, and Evaluation, 10*, 1-10. doi:10.7275/jyj1-4868

Luetters, C., Huang, M. H., Seeman, T., Buckwalter, G., Meyer, P. M., Avis, N. E., . . . Greendale, G. A. (2007). Menopause transition stage and endogenous estradiol and follicle-stimulating hormone levels are not related to cognitive performance: cross-sectional results from the study of women's health across the nation (SWAN). *Journal of Women's Health, 16*, 331-344. doi:10.1089/jwh.2006.0057

Randolph, J. F., Jr., Sowers, M., Bondarenko, I. V., Harlow, S. D., Luborsky, J. L., & Little, R. J. (2004). Change in estradiol and follicle-stimulating hormone across the early menopausal transition: Effects of ethnicity and age. *Journal of Clinical Endocrinology and Metabolism, 89*, 1555-1561. doi:10.1210/jc.2003-031183

Timmerman, M. E., & Lorenzo-Seva, U. (2011). Dimensionality assessment of ordered polytomous items with parallel analysis. *Psychological Methods, 16*, 209-220. doi:10.1037/a0023353
